# Regulatory T Cell Attracting Therapy Accelerates Skeletal Muscle Functional Recovery Following Injury

**DOI:** 10.21203/rs.3.rs-7237053/v1

**Published:** 2025-08-22

**Authors:** Matthew A. Borrelli, Jordan JP. Warunek, Steven R. Little, Heth R. Turnquist

**Affiliations:** University of Pittsburgh; University of Pittsburgh School of Medicine; University of Pittsburgh; University of Pittsburgh School of Medicine

**Keywords:** Skeletal Muscle, Treg, Injury, Microparticles, Tissue Repair, Immunomodulation

## Abstract

Skeletal muscle injuries are a common consequence of physical activity, repetitive movements, and trauma. Regulatory T cells (Treg) have recently been identified as critical mediators of immune repair response after injury, and treatments effectively targeting Treg may accelerate injury resolution. CCL22 is a chemokine that recruits CCR4-expressing cells, particularly Treg, to sites of inflammation or immune regulation, such as tumor microenvironments. When a sustained release formulation of polymeric microparticles (MP) delivering CCL22 (CCL22MP), was administered after cardiotoxin (CTx)-mediated muscle injury, significantly improved limb function was observed on days 3 and 5 post injury. Histologic evaluation of the injured limbs showed reduced area of injury in CCL22MP treated limbs. Analysis of the local immune populations revealed augmented Treg concentrations, as well as increased myeloid derived suppressor cell and neutrophil frequency. These findings reveal that amplifying local Treg to damaged areas improves outcomes, thus offering a translationally promising approach after muscle injury.

## INTRODUCTION

Skeletal muscle injuries are a common and often unavoidable consequence of prolonged physical activity, intense exercise, and trauma. Muscle damage encompasses over 40% of all sports and exercise-induced injuries, and nearly 20% of injuries sustained during basic training of armed forces^1^. Among the general population, more than 21 million Americans over the age of 18 seek medical care for muscle injuries each year^2^, amounting to hundreds of billions in lost yearly productivity^3^. Muscle injuries of differing severity occur when myofibers undergo structural damage leading to rupture or tear, resulting in swelling, pain and lost limb function^4^. Muscle injuries are often treated through a combination of rest, ice, compression elevation (RICE) and broad spectrum anti-inflammatory medications, such as non-steroidal anti-inflammatory drugs (NSAIDs) or corticosteroids^4^. While NSAIDs and corticosteroids can reduce pain and swelling associated with inflammation, they also impair critical functions of the infiltrating immune cells that remove cellular debris and promote regeneration^5^. For this reason, non-specific anti-inflammatory therapeutics often have poor efficacy and, in some cases, impair muscle regeneration^6,7^. This has led to growing concerns about the overuse of NSAIDs for post-injury management in physically active populations^8^.

Single-cell and spatial transcriptomics, precise lineage tracing and cell-specific reporter constructs have enabled a detailed understanding of the mechanisms underlying the immune responses to skeletal muscle injury and how individual cell types contribute to the repair, remodeling and resolution of the injured tissue^4,5,9–16^. Damage to the myofiber bundles can lead to necrosis, or inflammatory cellular death, of myocytes, which contributes to the release of damage associated molecular patterns (DAMPs), pro-inflammatory cytokines^17^, and chemoattractant molecules (chemokines), such as MCP-1 (CCL2), MIP-1α (CCL3), and MIP-1ß (CCL4)^18^. These chemokines signal through CCR2 and CCR5 receptors to attract neutrophils and monocytes to the injured tissue^18^. There is some evidence that they also act directly on myocytes to initiate proliferation^18,19^. Attracted neutrophils provide necessary clearance of cellular debris^16^ while monocytes differentiate into inflammatory Ly6C^hi^ macrophages that are critical for early satellite cell activation, myogenesis, and preventing maladaptive fibrosis^20^. While engulfing cellular debris from the injured tissue, macrophages gradually receive signaling to differentiate into Ly6C^lo^ macrophages between day 3 and 6 post-injury^21,22^, which exhibit a reparative phenotype^20^, and begin orchestrating tissue remodeling^10,11,23^. Specifically, they coordinate myogenesis and tissue remodeling through extracellular matrix deposition and stimulating tissue vascularization^24^. Importantly, effective tissue repair requires a balance of pro-inflammatory and reparative pathways, as chronic influx or overexuberant activity of these early inflammatory cells also have the potential to delay or impair the tissue repair. Situations may occur where monocytes differentiate into osteoclasts and reduce bone density^9^ or collateral damage by neutrophils and their release of NETs can damage healthy cells^16^. However, countering chemoattraction of neutrophils and monocytes via antiserum blockade of CCL2 or genetic ablation of CCR2 and CCR5 receptors results in severely diminished repair^18^. Additionally, early addition of the anti-inflammatory cytokine, IL-10, can also impair skeletal muscle regeneration by inhibiting macrophage proliferation and necessary cytokine expression^22^. Precise regulation is required to facilitate appropriate expansion of the macrophage population while avoiding the negative consequences of a chronic or overexuberant response.

Regulatory T cells (Treg) are recognized endogenous controllers of inflammatory immune cells that prevent autoimmunity and maintain tissue homeostasis^13,14,25^. Treg utilize several immunomodulatory mechanisms to suppress inflammatory responses, including the secretion of immunomodulatory proteins (IL-10 and TGFβ)^26^, scavenging IL-2, which is needed to perpetuate effector T cells^27^, and controlling the co-stimulation available to T cells^28^. Treg can also exert control over neutrophils^25^ and macrophages^14^ to reduce their production of inflammatory cytokines. It has also emerged, however, that Treg are not only immunosuppressive, but can be programmed by local injury signals to fulfill important reparative functions^14^. After injury to skeletal muscle, as well as other tissues^29–31^ and organs^32–35^, Treg detection of interleukin-33 (IL-33) leads to their proliferation and release of reparative effector molecules, such as IL-13, a cytokine that polarizes local monocytes and macrophages towards a pro-repair subsets^33,36^. IL-33 also stimulates Treg to produce potent growth factors, such as the epidermal growth factor amphiregulin (Areg) or vascular endothelial growth factor A (VEGF), among others^14,37,38^. Treg production of Areg is a particularly important mechanism for muscle repair, as Areg directly stimulates satellite cell proliferation after injury^14^. IL-33-mediated Tregs production of Areg has been shown to be of importance in other models of tissue injury including transplantation^39^, acute lung injury^33^, and myocardial infarction^34^. Treg repair functions are critical after injury, as global depletion of Treg following injury results in failed repair marked by lingering inflammation and disrupted reparative macrophage differentiation^14^. Unfortunately, Treg in injured muscles are of limited frequency, especially in aged individuals^14,38^, and slow to infiltrate or expand following injury^12^. Likewise, severe traumatic injuries cause the excessive release of inflammatory mediators that can cause inordinate inflammation and injury that overburden local reparative cells^40^.

Novel therapies able to limit inflammation, while promoting repair are thought to be especially promising. Given their function in regulating immune cell inflammatory activity and ability to stimulate direct and indirect tissue repair, Tregs have been gaining increasing interest as a potential therapeutic target in skeletal muscle injury^41^. We have developed a Treg-attracting formulation of microparticles capable of directing Treg chemotaxis to the site of administration via the controlled release of encapsulated CCL22^42^. CCL22 is a chemokine primarily involved in recruiting CCR4-expressing cells, particularly Tregs^43,44^, as well as Th2 cells, and some myeloid cell subsets, to sites of inflammation or immune regulation^45^. CCL22 plays a critical role in maintaining immune homeostasis, promoting anti-inflammatory responses, and has been implicated in tumor immune evasion^43,46–48^ and transplant tolerance by facilitating Treg accumulation^49,50^. CCL22 releasing microparticles (CCL22MP) have been employed successfully to control inflammation causing or perpetuating disorders including periodontitis^51^, dry-eye disease^52^, and transplantation rejection^49^, but CCL22MP have not yet been evaluated for their ability to improve regeneration after muscle injury by attracting Treg. As such, we assessed if the regulatory and direct regenerative functions of Treg could be harnessed for accelerated repair following acute skeletal muscle injury using CCL22MP treatment. Using a murine model of skeletal muscle injury, we clearly show that CCL22MP treatment is a highly promising and novel therapeutic that enhances functional strength recovery by rapidly amplifying the presence of Treg in the injured limbs to stimulate regeneration and reduce the area of injury.

## RESULTS

### Development and characterization of CCL22 releasing microparticles

**Figure 1:**
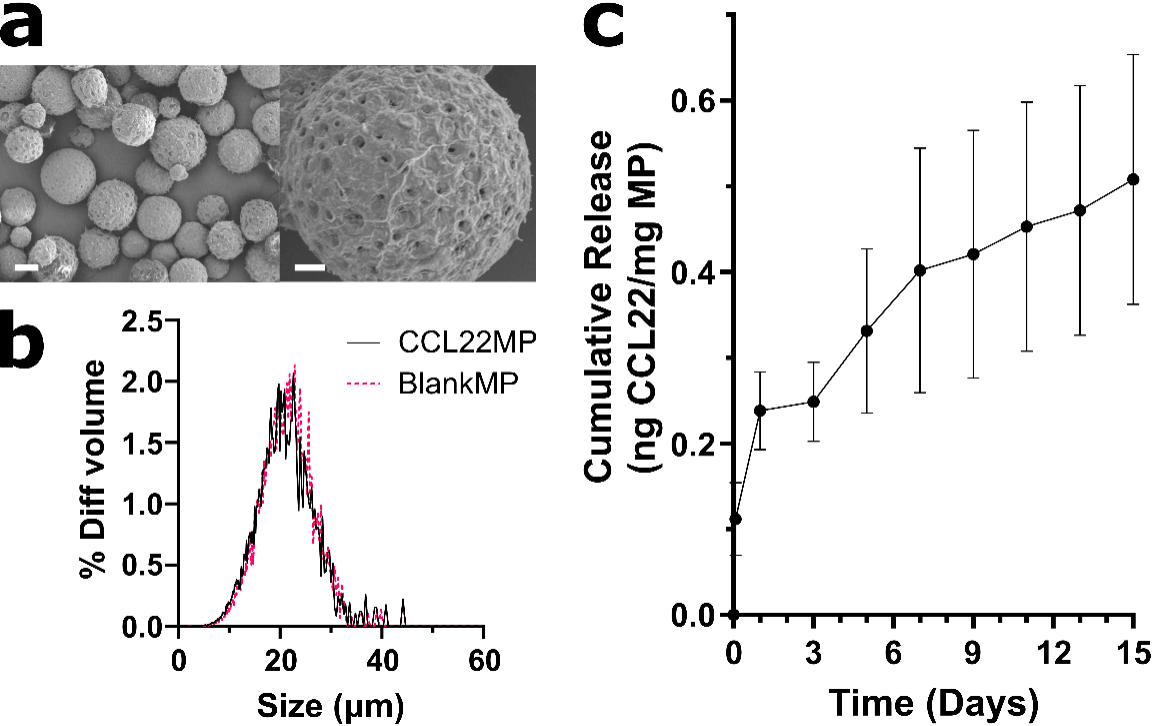
CCL22 encapsulated in PLGA MP releases for 15 days Proposed Treg attracting microparticles designed to release CCL22 protein were characterized. **a)** SEM images of 55 wt.% polyethylene glycol (PEG) terminated poly(lactic-co-glycolic) acid (balance RG502H PLGA) microparticles (MP) show spherical morphology with a rough, porous surface. Left – 800x, scale bar = 10 μm; Right – 3500x, scale bar = 3 μm. **b)** Particle size distributions of unloaded MP (BlankMP) and MP loaded with 5 μg of CCL22 (CCL22MP) show particles are between 15–25 μm in diameter. **c)**Quantification of CCL22MP release shows sustained and controlled release of CCL22 for a period of at least 15 days. Data shown represents the mean ± SD for N = 3 independent experiments.

Microparticles (MP) formulated with a blend of PEG terminated PLGA and RG502H, in which the PEG-PLGA fraction was gradually increased, were loaded with CCL22 (“CCL22MP”) or water (“BlankMP”) and 15 mOsm of NaCl (**Supplementary Fig. 1**). 55% PEG-PLGA (Balance RG502H) was selected for functional characterization and use *in vivo* due to its favorable release kinetics. More specifically, the release profile for this formulation was compatible with our desire to promote rapid and accelerated Treg infiltration by simulating an initial boost in chemotactic signal followed by sustained signaling to establish a stable chemotactic gradient. Scanning electron microscopy (SEM) imaging shows that both CCL22 and BlankMP had expected spherical morphology and that the surface is populated with pores to facilitate early release as reported previously^42^ (Fig. 1A). Both formulations show similar size distributions, in which the mean diameter ranges from 15–25 μm (Fig. 1B). The cumulative release profile of CCL22 from CCL22MP was quantified for 15 days (Fig. 1C). Following an initial rapid burst release of 0.2 ng CCL22/mg of MP, this formulation produces a sustained and controlled rate of release amounting to 20 (pg of CCL22)/(mg of MP)/(day) for 15 days.

### CCL22 releasing microparticles improve limb function following acute muscle injury

**Figure 2:**
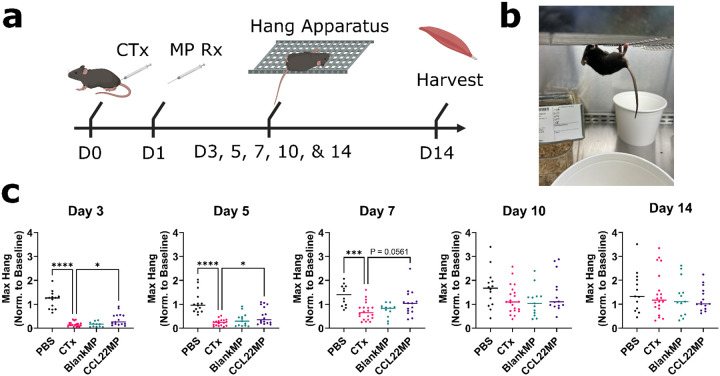
CCL22MP treatment improves limb function **a)** Experimental scheme – MP treatments were administered 24 hours after injury with limb function assessments evaluated on days 3, 5, 7, 10, and 14 via inverted cage hanging. **b)** Example image of a mouse hanging on an inverted cage. Additional images and video are contained in the supplemental data. **c)** The normalized hang duration (minutes) was significantly elevated in CCL22MP treated mice for days 3 and 5. Data shown represents mean ± SD of N = 14 to 22 mice. One-way anova was performed with Dunnet’s multiple comparisons correction to determine differences between each group and the CTX only control. * p < 0.05, *** p < 0.001, **** p < 0.0001.

The impact of CCL22MP treatments were evaluated in a variant of the well characterized cardiotoxin (CTx)-induced muscle injury model^53,54^. Specifically, mice received a single injection of CTx in PBS (“CTx”) or PBS alone (“PBS”) into the gastrocnemius of the right and left hindlimb and the extensor carpi of the forelimbs to generate injury to all extremities. 24 hours post injury, a subset of mice received intramuscular injections of CCL22MP suspended in PBS (“CCL22MP”) to the injured muscles, the CTx subsets received a control treatment of PBS alone, and others were treated with empty or “BlankMP” suspended in PBS. Limb function was then quantified on days 3, 5, 7, 10, and 14 following injury induction (Fig. 2A). Here, limb function and motor coordination were tested using the inverted wire hang method (reference image in Fig. 2B). Hang duration was normalized to baseline measurements taken prior to CTx injury and plotted in Fig. 2C. As expected, mice in the uninjured PBS treated control group did not display a functional defect. Mice in the CTx group that were administered CTx and then treated with PBS exhibited significantly reduced functions compared to PBS only controls at days 3, 5, and 7 (Fig. 2C). Mice administered CTx and then treated with CCL22MP had significantly improved hang duration at day 3 and 5 timepoints, relative to the CTx injury group. By the day 7 timepoint, hang duration for CCL22MP treated mice remained elevated, but other control groups began to show increasingly improved performance. By day 14 post-injury, all groups exhibited a return of function to baseline measurements. Thus, CCL22MP accelerates functional strength and recovery during the acute phase following muscle injury.

### CCL22MP treated limbs exhibit reduced injury in CTx treated skeletal muscles

**Figure 3:**
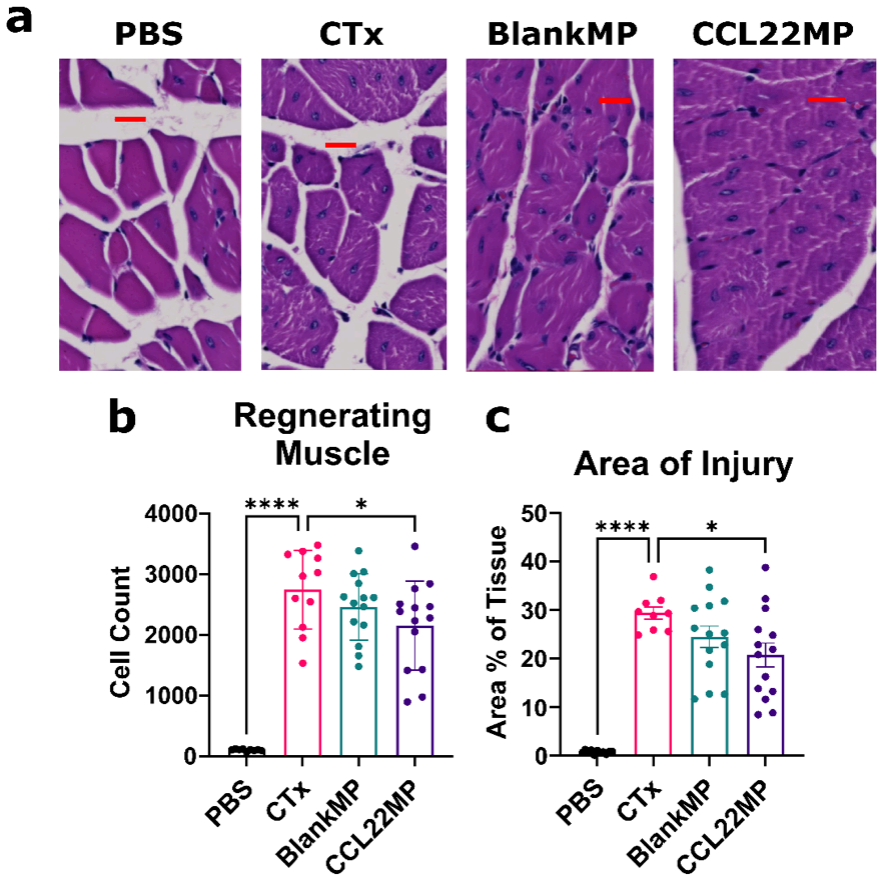
CCL22MP reduces area of injury Histologic evaluation of hindlimb muscles harvested on day 14 post-CTx, following functional measurements. **a)** Representative H&E staining – Scale bar (red) is 20 μm. **b)** Cell count of regenerating (centrally nucleated) myofibers within tissue sections. **c)** Area % of regenerating muscle. Data shown represents mean ± SD for N = 14 mice and each data point is the average of both hindlimbs. One-way Anova was performed with Dunnet’s multiple comparisons correction to determine differences between each group. * p < 0.05, **** p < 0.0001.

To define how CCL22MP-mediated improvements in function relates to skeletal muscle injury repair, we completed histologic assessment of cross-sectioned gastrocnemius muscles at day 14 post-CTx administration. Figure 3A shows representative images of the regenerating muscle for each treatment group. PBS alone tissues exhibit normal muscle architecture consisting of well-organized and tightly packed fibers with peripheral nuclei as expected. CTx treated muscles, however, displayed disrupted and irregularly shaped fibers with centrally located nuclei (Fig. 3A). In samples from CCL22MP-treated mice, the diameter of the myofibers appear increased relative to CTx control. Myofiber cross-sections from BlankMP-treated limbs exhibited an increased frequency of peripheral nuclei, which corresponds to the final phase of regeneration. Quantification of the total number of centrally nucleated, regenerating myocytes and their area percentage relative to the entire gastrocnemius is presented in Fig. 3B–C. CCL22MP treatment results in a significant reduction in the number and area of regenerating cells, relative to the CTx injury control. Taken together, CCL22MP treatment improves muscle regeneration and reduces injury more effectively than BlankMP, supporting its reparative potential after muscle injury.

### CCL22MP enrich for immunoregulatory cell populations at the site of injury

**Figure 4:**
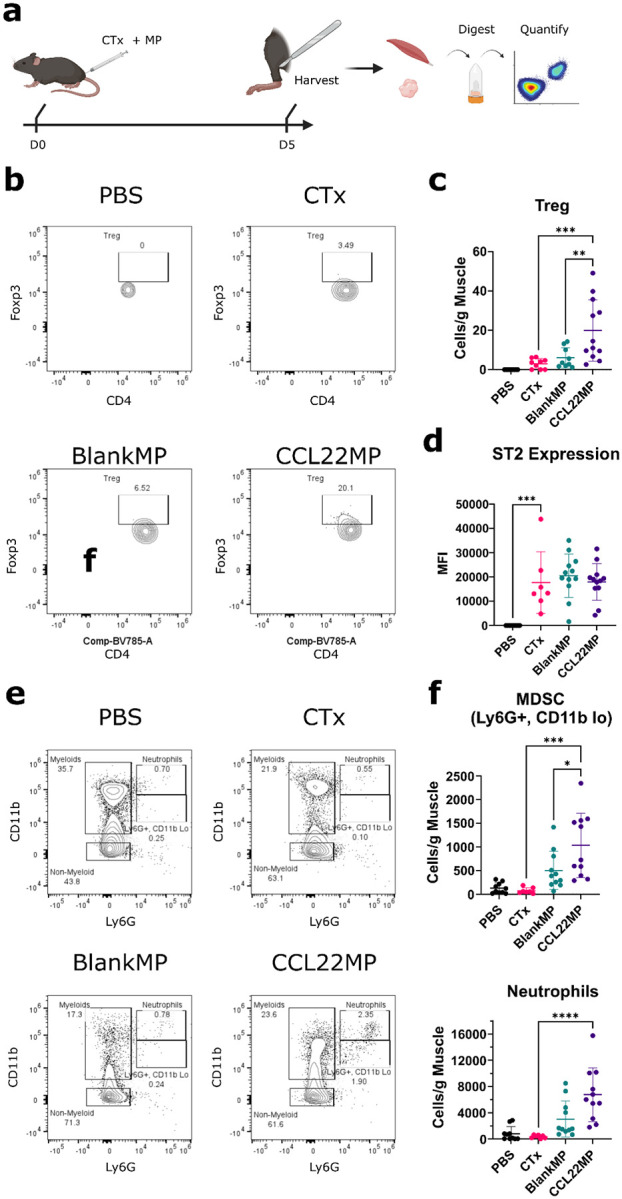
CCL22MP treatment augments Treg and myeloid cells within the injured muscle **a)** Experimental scheme – Mice were co-administered microparticle (MP) therapeutics and cardiotoxin (CTx); after 5 days the limb muscles and popliteal draining lymph node were harvested, digested, and leukocytes were isolated. **b)** Representative quantitation of Foxp3^+^ CD3^+^ CD4^+^ CD25^+^ Treg population in the CD45^+^ B220^−^ CD11b^−^ CD11c^−^ gate is shown **c)** Quantification of the number of Tregs present in the treated limbs. **d)** Assessment of ST2 expression on Treg. **e)** Representative assessment of myeloid populations from muscle digests. CD45^+^ B220^−^ CD3^−^ CD11b^+^ gated cells is shown. **f)** Quantification of the number of CD11b^+^ Ly6G^hi^ neutrophils and CD11b^+^ Ly6G^lo^ MDSCs in each group. Data shown is mean ± SD and represents the aggregate digest of both hindlimbs for N = 9 to 12 mice per group. One-way Anova was performed with Dunnet’s multiple comparisons correction to determine differences between each group. * p < 0.05, ** p < 0.01, *** p < 0.001, **** p < 0.0001.

We next defined how CCL22MP treatment shapes immune responses following CTx injury using multispectral flow cytometry on immune cells isolated from the skeletal muscles and nearest draining lymph nodes (see **Supp.** Figure 2 for a representative gating strategy). In these studies, mice were injected with CTx or PBS and then administered CCL22MP in PBS, BlankMP in PBS, or PBS alone (Fig. 4A). Assessment of CD45^+^ leukocyte populations isolated from the injured gastrocnemius muscle 5 days after injury and treatment reveals an increased frequency of CD3^+^ CD4^+^ CD25^+^ cells expressing the Treg transcription factor Foxp3 for CCL22MP treated limbs (Fig. 4B **and Supplementary Fig. 4A**). The total number of Tregs normalized to the mass of hindlimb tissue was calculated and also revealed a significant increase relative to the BlankMP or CTx alone control groups (Fig. 4C). The level of expression of the IL-33 receptor, serum stimulation-2 (ST2), was increased on local Foxp3^+^ CD3^+^ CD4^+^ CD25^+^ by muscle injury, however, there was not a difference noted between treatment groups (Fig. 4D). Further assessment of the myeloid compartment found that there was an elevated frequency of rare CD45^+^ Ly6G^+^ CD11b^Lo^ cells that are phenotypically consistent with those described as monocyte-derived suppressor cell (MDSC)^55^ (Fig. 4D–E and **Supp.** Figure 3A). There was also an elevated frequency of neutrophils in the CCL22MP treated limbs compared to CTx alone (Fig. 4D and **Supp.** Figure 3A). Quantification of the total number of MDSC and neutrophils showed a significant increase in response to CCL22MP treatment relative to BlankMP-treated limbs (Fig. 4E). We did not detect significant differences in the frequency of intramuscular B cells, dendritic cells, as well as macrophages (**Supp.** Figure 3), however, intramuscular B cells and macrophages exhibited a trend towards increased frequency (**Supp.** Figure 3) and number (**Supp.** Figure 4) with CCL22MP treatment. To better understand changes in the local immune populations supporting tissue healing versus any treatment-induced distant immunomodulatory impacts, we assessed CD45^+^ leukocytes from the draining popliteal lymph nodes. Similar flow cytometric analysis found minimal elevations in the frequency of immune cells assessed (**Supp.** Figure 4). CCL22MP treatment was associated with increased numbers of macrophages in the draining LN (**Supp.** Figure 4F). There was not an increase in the frequency of Treg, nor did we observe changes in ST2 in the draining lymph nodes (**Supp.** Figure 4A). In total, these findings demonstrate that CCL22MP treatment enhances local accumulation of Tregs, presumed MDSC, and neutrophils at the site of muscle injury, which was associated with an accelerated restoration of muscular function.

## DISCUSSION

The present study reports how enhancing the local population of Tregs through the administration of a Treg-attracting microparticle formulation affects the recovery of injured skeletal muscle. Prior studies have delineated that Treg play critical roles in skeletal muscle injury^13,14,38^ by not only regulating infiltrating leukocytes^25^, but also secreting growth factors like Areg that stimulates muscle satellite cell proliferation^14^. Despite increasing interest in leveraging Tregs as a therapeutic target in skeletal muscle injury^41^, to our knowledge, there have not been prior studies that have investigated the amplification of Tregs through local drug delivery in the injury site as a potential treatment. In this first-of-its-kind study amplifying the local population of Tregs in the injured muscle, we found that treatment with microparticles releasing CCL22 (CCL22MP), a Treg chemoattractant, significantly improved the limb function of mice at early timepoints. Subsequent investigations revealed a reduced area of injury in the limb, as well as significantly elevated amounts of Treg, presumed MDSC, and neutrophils, locally, but not in the adjacent lymphoid tissues. Together, these findings establish a novel and effective strategy for modulating the immune microenvironment at the site of injury through local Treg recruitment, highlighting the therapeutic potential of CCL22MPs in accelerating muscle repair and functional recovery.

The importance of Treg chemoattraction to local immunomodulation was originally defined in studies developing an understanding how tumors evade immune clearance^43,46–48,56^. These studies revealed that the chemokine, CCL22, is secreted by tumor associated macrophages and dendritic cells^46–48^. This directs Treg that are enriched for the CCL22 receptor, CCR4, to infiltrate the tumor site^43,56^. Taking inspiration from this, we have developed a PLGA microparticle-based platform that has demonstrated the ability to attract adoptively transferred Tregs to the CCL22MP injection site^42^. Subsequent studies demonstrated CCL22MP treatment to be efficacious in models of inflammatory disease including periodontitis^51^, dry-eye disease^52^, and transplantation rejection^49^. In each of these studies, RG502H – an acid terminated PLGA polymer containing equal ratio of glycolic and lactic acid repeating units – was utilized as the encapsulating polymer. CCL22 release from this polymer formulation has produced linear release profiles that delivered 80–4500 pg CCL22/mg of MP by day 14, which is compatible with Treg functional immunobiology. PLGA polymer is one of the most widely explored degradable polymers for delivery of protein therapeutics because of its biocompatible degradation products, glycolic and lactic acid, and its prior use in FDA-approved formulations^57^. Recently, PLGA polymers with various terminal groups have become more accessible^58^ and, in some cases, facilitate a gain of function such as improved cellular targeting of nanoparticles^59^ or reduced phagocytic clearance^59,60^. For the current study, we included poly(ethylene glycol) (PEG) terminated PLGA in the encapsulating polymer due to its ability to confer several benefits. Specifically, PEG has been reported to slow the clearance rate of polymeric particles^60,61^, and, following injury, it has been demonstrated to provide membrane sealing^62^ and to reduce apoptosis^63^. The resultant formulation of 55 wt.% PEG-PLGA and 45 wt.% RG502H produced linear release behavior amounting to 500 pg of CCL22/mg of MP delivered by day 14, which is consistent with our previously reported release profiles that produced rapid augmentation of Treg at the site of injury. That we did not witness systemic changes in Treg suggests precisely controlled local delivery.

The importance of a well-coordinated immune response in skeletal muscle regeneration has been routinely characterized using murine models, including CTx injections. The regenerative program after CTx administration depends on a temporally regulated sequence of events, beginning with an acute inflammatory phase dominated by pro-inflammatory macrophages, followed by a reparative phase characterized by restorative macrophages^53^. However, Tregs also play critical roles in shaping skeletal muscle repair. Recent work investigating Tregs functions in muscle repair documented a subset of skeletal muscle-resident Treg that highly express the helios transcription factor and the neuropilin transmembrane receptor^14,38^. Their helios and neuropilin expression indicate that these Tregs are most likely thymic derived and not peripherally induced. These Treg were also reported to highly express ST2 receptor and respond to IL-33 with production of Areg^14,38^. Transgenic mice where Treg lack Areg were exploited to determine that Areg contribute a significant role in shaping the Treg response to skeletal injury by stimulating satellite cell proliferation^14,38^. While Areg has also be implicated in suppressive functions^64^, several studies using Treg lacking Areg have found that systemic immune responses to virus^65^ and alloantigen^39^ are not modified in the absence of Treg-expressed Areg. Tregs typically begin to amass in the injury site 4 days following initial injury via CCL3 chemoattraction^12^. Thus, CCL22MP treatment one day following injury has the potential to augment and accelerate the normal attraction of Treg to injured skeletal muscles. Indeed, our experiment administering CCL22MP after CTx-mediated injury resulted in a significant improvement to limb function on post-injury days 3 and 5 – timepoints where Treg are typically only just arriving. Consistent with improved function, subsequent histologic analysis on post-injury day 14 showed that CCL22MP treated limbs have reduced injury area, suggesting that CCL22MP treatment facilitated a reduction in damage spreading or accelerated repair relative to the control group. Whether this is the result of Areg-mediated repair or local immunomodulation by attracted Treg, however, will require further investigations using precise transgenic mice allowing the targeting of Areg or molecules implicated in Treg immunomodulation. BlankMP show a slight reduction in the injury area, though it is not statistically significant, indicating the PEG may be providing an anti-apoptotic effect, which would be consistent with prior reports^62,63^. Additionally, lactic acid has beneficial effects following muscle injury^66^. Thus, lactic acid degradation products from locally injected microparticles may also be promoting the observed differences in the BlankMP-treated limbs. These effects would be conserved for CCL22MP treatment and may be providing a synergistic benefit that complements the effects of attracted Treg.

Our flow cytometry assessments define how CCL22MP influenced the immune populations in the injury site and the draining lymph nodes, Treg in the injury site, but not the nearest draining lymph node, were significantly expanded for mice receiving CCL22MP. This result indicates that CCL22MP exerts a targeted effect, enhancing Treg recruitment or retention specifically within the tissue microenvironment of the injury, without inducing systemic or regional immune changes. This localized immunomodulation is predicted to be therapeutically advantageous because it will enhance repair and suppression at the site of injury, while minimizing any risk of systemic immunosuppression. Notably, there was an increased expression of ST2 on Treg at the site of injury, but no difference in the ST2 expression among the treatment groups. This suggests that ST2 expression is a response to the injury microenvironment itself, independent of CCL22MP treatment. This aligns with our recent studies showing that ST2^+^ Treg emerge upon infiltrating in inflamed or damaged tissues, where IL-33 is often released by stressed or necrotic cells^39^. Thus, the injury environment, not CCL22, appears to be sufficient to induce this ST2, and presumably allow IL-33 to support the reparative Treg phenotype.

The changes to B cell and macrophage populations identified in the hindlimb and lymph node are largely not statistically significant, but do suggest these populations are attracted or expanded in response to BlankMP and CCL22MP treatments. It is possible that the microparticles caused a foreign body response in the hindlimbs. It is well known that large microparticles (> 20–30 μm^67,68^) are not readily phagocytosed resulting in a state of frustrated phagocytosis^69^ and activating macrophages^70^. Notably, we did observe CCL22MP treatment caused an expanded Ly6G^+^ CD11b^Lo^ myeloid cell and neutrophil populations. The Ly6G^+^ CD11b^Lo^ phenotype is associated with the monocyte derived suppressor cell phenotype, which possess potent immunosuppressive and reparative capabilities in injury^10,11,23^. The increase in neutrophil population 5 days following injury was unexpected. These cells typically undergo apoptosis following clearance of cellular debris 2–3 days after injury^15^. However, *in vitro* studies have shown Tregs can act to reduce neutrophil IL-6 expression and skew them towards a suppressive and pro-repair phenotype that secretes IL-10 and TGF-ß, heme oxygenase-1, and indoleamine 2,3-dioxygenase^25^. Thus, it is possible that the attracted Treg are sustaining the neutrophil population to perform anti-inflammatory and pro-repair roles. Another possible explanation is that these neutrophils and MDSCs are co-migrating into the injured limb in response to CCL22. Neutrophils have been reported to express an array of chemokine receptors including CCR1, CCR2, CCR3, CCR5, CXCR1, CXCR2, CXCR3, and CXCR4, and that the expression of these receptors differs among injury infiltrating neutrophils and peripheral blood neutrophils^71^. Of interest is CCR2 expression, which is the target receptor or CCL2. Interestingly, CCL2 can also bind to CCR4^72^; because CCR4 is the target receptor for CCL22, it is possible CCL22 could bind to the CCR2 receptor, reciprocally. Further, it has been reported that chemokines can interact with one another to produce synergistic migratory effects^73^. For instance, in the context of allergic dermatitis, CCL22 was shown to interact with CXCL10 resulting in amplified migration of CCR4 + T cells into the tissue^74^. A similar phenomenon has been observed for neutrophil migration, in which CCL2 and CCL7 synergize with CXCL8 to enhance their migration during acute respiratory distress syndrome^75^.

The present study provides insights into how localized regulatory T cell amplification following muscle injury can affect limb functional recovery. One important limitation of this work is that it lacked quantification of secreted reparative, inflammatory, and anti-inflammatory factors from the local immune populations. Logically, increased numbers of Treg that are phenotypically identical should result in a proportional increase in secreted factors. However, it is possible that the activity of the expanded Treg population is also increased. Likewise, the studies completed did not quantify how the expanded Treg population affects the local balance of inflammatory (e.g. IFNγ, TNFα, IL-6, etc.) and anti-inflammatory (e.g. TGFß, IL-10, etc.) signals. Thus, future experiments that investigate how CCL22MP treatment may alter these secreted factors following muscle injury are merited. Further, this study lacks a full investigation into the expanded neutrophil population observed in CCL22MP treated limbs. Future studies could investigate the neutrophil populations to inform what role they are playing in the injured limb. Another limitation of this work, however, is a lack of precise understanding of the mechanism these Treg improved functional repair. Without blockade of the reparative effectors, IL-13 and Areg, secreted from Treg or the suppressive mechanisms of these cells, such as IL-10 or CTLA-4, it is unclear what their individual contributions are to the observed functional recovery and reduction to injury. Most likely benefits require both suppressive and reparative Treg functions. Therefore, future studies employing the Cre-loxP system to alter genetic expression of the repair and suppressive genes in Foxp3^+^ Treg in this injury model with CCL22MP treatment may provide greater insight. Additionally, this study focused on a CTx model of muscle injury which does not interfere with basal repair functions. While the improvement that CCL22MP treatment conferred was considerable in this model, repeating this work in injury models that have limited regenerative capabilities, such as the volumetric muscle loss model, or in mice that express poor or dysregulated muscle regeneration, such as mdx mice that exhibit muscular dystrophy, may provide a greater understanding of the potential benefits of this treatment. Despite these limitations, the work reported in this manuscript has broad implications for the field of regenerative medicine. Because the immune response is critical to tissue repair and remodeling, this Treg attracting treatment has potential to be beneficial in other injury and regenerative models.

In total our study establishes that CCL22-enriched Tregs have a significant ability to shape the injury microenvironment. Intervening within the first 24 hours of an acute skeletal muscle injury with a Treg-attracting microparticle formulation resulted in early improvements to limb function and a reduction in total injury. This study is the first of its kind to manipulate Tregs in the injury site, directly, and may provide similar efficacy in other models of muscle injury and trauma. Future work exploring how early intervention with Treg-targeting therapies affects the cellular microenvironment following injury in muscles and other tissue types will be insightful and may lead to the discovery of new therapeutic targets or establish if local Treg could be replaced by controlled release of their delivered reparative or immunoregulatory effectors.

## METHODS

### Materials:

#### Polymers:

RG502H Poly(D,L-lactic-co-glycolic acid) (PLGA) polymer, with 50:50 lactide:glycolide composition was obtained from Evonik (Essen, Germany). Poly(ethylene glycol) (PEG) terminated PLGA (PEG-PLGA) and Poly(vinyl alcohol) (PVA, 98 mol% hydrolyzed, *M*_*w*_ = 25000 g mol^− 1^) were purchased from PolySciences (Warrington, PA).

#### Protein:

Carrier-free recombinant murine CCL22 (mCCL22) was obtained from R&D systems (Minneapolis, MN). Naja pallida cardiotoxin (CTx) was obtained from Sigma-Aldrich (St. Louis, MO) and diluted to a working concentration of 0.03 mg/mL prior to injection. Bovine Serum Albumin (BSA) was obtained from Fisher Chemical (Waltham, MA).

#### Reagents:

Dichloromethane (DCM), Acetonitrile (ACN), Sodium Dodecyl Sulfate (SDS), was obtained from Fisher Chemical (Waltham, MA)

#### Cell Reagents and Buffers:

Phosphate buffered saline (PBS), fetal bovine serum (FBS), Hanks’ balanced salt solution (HBSS), Roswell Park Memorial Institute 1640 medium (RPMI), Cytiva percoll centrifugation media, and eBioscience Foxp3 / Transcription Factor Fixation/Permeabilization buffer from Fisher Scientific (Waltham, MA). FACS and perm buffer were produced in house by supplementing PBS with 5% Fetal Bovine Serum (FBS) and 0.1% sodium azide; 1% FBS, 0.1% sodium azide, and 0.1% saponin; and 5% FBS, respectively.

#### Microparticle (MP) formulation:

Microparticles (MPs) encapsulating CCL22 or blanks were formulated via a double emulsion-evaporation method, as described in our previous work^42,76^. Briefly, MP formulation involves the formation of an emulsion between an inner, aqueous phase (200 μL vol) that contains CCL22 protein (5 μg) (CCL22MP) or water (BlankMP) and the outer, organic phase that contains PLGA polymer (200 mg, 55 wt.% PEG-PLGA & balance RG502H) solvated in dichloromethane (DCM) (4 mL). The aqueous phase for CCL22MP and BlankMP was formulated with 0.375 mg BSA to stabilize the encapsulated protein, and an osmolarity of 15 mOsm to produce surface pores. The resulting mixture of protein and polymer was sonicated (Active Motif EpiShear probe sonicator, 110V) at 55% amplitude for 10s to form the first emulsion (water-in-oil, w/o), and then transferred to a 2% PVA solution (60 mL) and homogenized (L4RT-A; Silverson, East Longmeadow, MA) at 3000 rpm for 1 minute, forming the second emulsion (w/o/w). Following homogenization, the solution was transferred to a 1% (w/v) PVA solution (80 mL) and stirred at 600 RPM for 3 hours on ice to allow the DCM to evaporate. Freshly formed MPs were centrifuged (Eppendorf 5810R 15 amp version, 200 xg for 5 min at 4°C) and washed four times (4x) with 4°C MilliQ water (Milli-Q IQ 7000, Millipore Sigma) (35 mL). The MPs were then re-suspended in MilliQ water (5 mL), flash-frozen with liquid nitrogen (5 minutes), and lyophilized (Benchtop Pro, Virtis SP Scientific) (< 100 mTorr, 48–72 hours).

#### Microparticle characterization and release assays:

Scanning electron micrographs of MPs were obtained using a scanning electron microscope (ZEISS Sigma500 VP), located in the University of Pittsburgh Nanofabrication and Characterization Core Facility. Size distributions of MPs were determined using volume impedance measurements on a Beckman Coulter Counter (Multisizer-3; Beckman Coulter; Brea, CA). *In vitro* release behavior was characterized by incubating 10 mg of MPs in 1 mL of release media (1% (w/v) bovine serum albumin (BSA) in PBS) and incubated on a roto-shaker (Thermo Scientific^™^ Tube Revolver Rotator, 1.5–2 mL Eppendorf tube paddles, speed 10) at 37°C. At specified time intervals, MP suspensions were centrifuged (580 g for 5 mins at room temperature) (Eppendorf 5417R), 800 μL of the supernatant was removed and replaced with 800 μL of fresh release media, and the MPs were resuspended and placed back on the roto-shaker. Supernatant concentrations of CCL22 were determined via enzyme-linked immunosorbent assay (ELISA; R&D Systems), and subsequently the ELISA results were interpreted through quantification of optical density (SpectraMax M5; Molecular Devices; Sunnyvale, CA). Release profiles generated from measured concentrations of CCL22 had any background signal from BlankMP subtracted and then normalized to the total amount loaded within MPs (quantified by solvating MPs and extracting the protein liberated) and MP mass.

#### CTx Muscle Injury Model & MP Treatment:

Limb injury was conducted following a variant of the well characterized cardiotoxin skeletal muscle injury model^53,54^. Mice (B6 Wildtype, Male, 8–10 wks old) were randomly assigned to one of 4 groups: Healthy control (PBS), injury and treatment control (CTx), Vehicle control (BlankMP), and treatment group (CCL22MP). Mice were anesthetized with 4% Isoflurane and injected with 50 μL of CTx (0.03 mg/mL) (CTx, BlankMP, and CCL22MP groups) or 50 μL of saline (PBS group) into each forelimb (Extensor Carpi) and hindlimb (Gastrocnemius). Injured limbs were administered 50 μL of CCL22MP (10 mg/mL) (CCL22MP group), BlankMP (10 mg/mL) (BlankMP group), or saline (CTx group) was injected into each forelimb and hindlimb. For functional studies, treatment occurred 24 hours after injury and for flow cytometry studies treatment occurred simultaneously with injury induction. At the conclusion of a study, mice (age: 8–16 weeks, weight: 22–30g) were euthanized via CO_2_ (99% purity) and subsequent cervical dislocation. The Gastrocnemius was then collected for further analysis.

#### Study approval and reporting:

All procedures completed at the University of Pittsburgh were approved by the IACUC of the University of Pittsburgh (protocol 22051178) and complied with the NIH’s Guide for the Care and Use of Laboratory Animals (National Academies Press, 2011). This study is reported in accordance with ARRIVE guidelines (https://arriveguidelines.org).

#### Limb function and histologic injury assessment:

At specified timepoints following CTx injury (POD 3, 5, 7, 10, & 14) limb function was assessed via the commonly used inverted wire hang method^77–79^. Briefly, mice were placed on a wire cage, the cage was inverted, and the time it took for the mice to fall off the cage was recorded. For each mouse, this assessment was repeated in triplicate with at least 60 seconds rest between each trial. The maximum value of the three trials was calculated and normalized to the maximum value measured during a baseline wire hang test performed prior to injury. The data was tabulated and evaluated for statistical differences to the CTx injury control. *Histology*: After the functional measures were completed on Day 14, the gastrocnemius muscles were collected for histologic analysis. The muscle tissue was fixed in 4% paraformaldehyde for 2 hours followed by a 2-step dehydration utilizing an overnight incubation in 30% sucrose and final storage in 70% ethanol (balance water). Samples were sectioned (10μm thick) at the midpoint of the gastrocnemius perpendicular to the origin and insertion, and hematoxylin and eosin (H&E) staining was then performed. Regenerating myofibers have centrally located nuclei and at later stages of regeneration and later mature into normal sized myofibers with peripheral nuclei^80^. Thus, quantification of the count and area of centrally nucleated, regenerating muscle cells was performed using QuPath open-source software^81^.

#### Tissue Collection:

##### Muscle:

C57BL/6 wildtype mice were euthanized and gastrocnemius skeletal muscle were carefully excised and then minced by scissors in RPMI 1640 digestion buffer (2% FBS, 12.5mg collagenase IV (Thermo Fisher; 17104019) and DNAse I (Sigma Aldrich; DN25–1G)). The tissue was further homogenized using Miltenyi gentleMACS Dissociator and incubated at 250 rpm and 37°C for 45 minutes. After digestion, homogenate was passed through a 70μm nylon mesh strainer and processed for mononuclear cells by using a 37.5% Percoll gradient (GE Healthcare). Pellets were resuspended in 2% RPMI, counted by hemacytometer and prepared for flow cytometry staining. *Lymph nodes*: The nearest draining lymph node (popliteal) to the gastrocnemius was collected simultaneously. Each replicate represents the aggregate collection of both popliteal lymph nodes collected from each limb. The collected lymph nodes were minced by scissors in RPMI 1640 and passed through a 70μm nylon mesh strainer. The cells were pelleted by centrifugation and resuspended in RPMI 1640 for counting and prepared for flow cytometry staining.

#### Flow Cytometry:

Harvested cells were incubated in 5% normal goat serum, stained with surface antibodies for 25 minutes on ice, washed and placed in Foxp3/Transcription Factor Staining Fixation/Permeabilization Buffer Set (eBioscience) overnight. For intracellular staining, cells were permeabilized (eBioscience) and stained with antibodies for 45 minutes on ice. Data was acquired using a Cytek Aurora spectral flow cytometer (Cytek Biosciences) and analyzed using FlowJo v10.9.0 (BD Life Sciences). The final stained samples were then washed 2x with FACS buffer and run on Cytek’s Aurora Cytometer (Fremont, CA).

#### Flow Cytometry Antibodies:

BUV496 rat anti-mouse CD45 (1:300 BD Biosciences Cat: 569673; Clone: 30-F11), Pacific Blue anti-mouse F4/80 (1:150 BioLegend Cat: 123124; Clone: BM8), Brilliant Violet 785 rat anti-mouse CD4 (1:200 BioLegend Cat: 100552; Clone: GK1.5), Brilliant Violet 650 rat anti-mouse CD86 (1:200 BD Biosciences Cat: 564200; Clone: GL1), Brilliant Violet 605 rat anti-mouse CD25 (1:200 BD Biosciences Cat: 563061; Clone: PC61), PE rat anti-mouse Ly6C (1:200 BD Pharmigen Cat: 560592; Clone: AL-21), PEcy5 rat anti-mouse B220 (1:200 BD Pharmingen Cat: 553091; Clone: RA3–6B2), Alexa Fluor 700 rat anti-mouse CD3 Molecular Complex (1:200 BD Pharmigen Cat: 561388; Clone: 17A2), APC/Fire 750 anti-mouse/human CD11b (1:200 BioLegend Cat: 101262; Clone: M1/70), BUV395 anti-mouse I-A/I-E (1:200 BD OptiBuild Cat: 743876; Clone: 2G9), BUV805 rat anti-mouse Ly6G (1:200 BD OptiBuild Cat: 741994; Clone: 1A8), Brilliant Violet 711 Hamster anti-mouse CD11c (1:200 BD Biosciences Cat: 563048; Clone: HL3), BUV737 rat anti-mouse IL33R (ST2) (1:100 BD OptiBuild Cat: 749323; Clone: U29–93), Brilliant Violet 421 rat anti-mouse CD8a (1:200 BioLegend Cat: 100737; Clone: 53 − 6.7), APC Armenian Hamster anti-mouse CD194 (CCR4) (1:100 BioLegend Cat: 131212; Clone: 2G12), FITC rat anti-mouse/rat FoxP3 (1:100 Thermo Fisher Cat: 11-5773-82; Clone: FJK-16s), PE-Cyanine7 iNOS rat anti-mouse antibody (1:400 Thermo Fisher Cat: 25-5920-82; Clone: CXNFT), Alexa Fluor 647 anti-mouse CD206 (1:200 BioLegend Cat: 141712; Clone: 068C2), PE-Dazzle 594 anti-mouse CD301b (1:300 BioLegend Cat: 146816; Clone: URA-1), PerCP/Cyanine5.5 rat anti-mouse T-bet (1:200 BioLegend Cat: 5760; Clone: 4B10), rat anti-mouse CD16/32 (TruStain FcX) (1:200 BioLegend Cat: 101320; Clone: 93), eBioscience Fixable Viability Dye eFluor 506 (1:500 Thermo Fisher Cat: 65-0866-14).

#### Statistics and reproducibility:

CCL22MP release experiments were performed in triplicate, and data represent means with standard deviation error bars. For multiple comparisons, One-Way Anova was performed followed by Šídák’s multiple comparisons test. Differences in means were considered to be significant if p ≤ 0.05.

## Supplementary Material

Supplementary Files

This is a list of supplementary files associated with this preprint. Click to download.

- SupplementaryFigures.docx

## Data Availability

Data associated with this manuscript is maintained on an internal data repository at the University of Pittsburgh. Data will be made available upon reasonable request to the corresponding author.
